# Postoperative Pituitary MRI Findings in Acromegaly: A Pictorial Review

**DOI:** 10.3390/diagnostics16162582

**Published:** 2026-08-15

**Authors:** Mahyar Daskareh, Fatemeh Abbasi, Negar Emamzadeh, Vahid Shahmaei, Mohammad Ghorbani

**Affiliations:** 1 Department of Radiology, Hospital of the University of Pennsylvania, Philadelphia, PA 19104, USA; 2 Radiology Department, Ziaeian Hospital, Tehran 1366736511, Iran; 3 Department of Medicine, Islamic Azad University, Tonekabon 4684161167, Iran; abbasi.vf2020@gmail.com; 4 Clinical Research Development Unit of Akhtar Hospital, Shahid Beheshti University of Medical Sciences, Tehran 1964714953, Iran; 5 School of Medicine, Iran University of Medical Sciences, Tehran 14149614535, Iran; negar.emamzade@gmail.com; 6 Radiology Technology Department, School of Allied Medical Sciences, Shahid Beheshti University of Medical Sciences, Tehran 1971653313, Iran; vahidshahmaei.sbmu@gmail.com; 7 Orthopedic Research Center, Department of Orthopedic Surgery, Mashhad University of Medical Sciences, Mashhad 9137913131, Iran

**Keywords:** acromegaly, transsphenoidal, MRI, postoperative pituitary MRI

## Abstract

Acromegaly is almost always caused by a growth hormone-secreting pituitary adenoma, and long-term cure depends on complete surgical resection and accurate detection of residual or recurrent disease on surveillance imaging. This pictorial review focuses on the 3–6-month postoperative interval, when routine surveillance MRI is commonly performed and dynamic contrast-enhanced pituitary imaging can be correlated with IGF-1 and GH trends. We first reviewnormal pituitary anatomy and imaging characteristics, and the typical location and signal characteristics of somatotroph adenomas, as a baseline for postoperative interpretation. Using representative cases of surgically treated acromegaly, we then illustrate the key postoperative MRI appearances on high-resolution T1- and T2-weighted and dynamic contrast-enhanced sequences. Benign patterns include non-enhancing T1-hyperintense packing material, thin rim or septal enhancement around thesurgical cavity, sheet-like floor-based granulation tissue, and simple stalk deviation with nromal rangecaliber, often seen in biochemically cured patients. High-risk imaging patterns comprise nodular or thick enhancing tissue in the resection bed, new or progressive cavernous sinus soft tissue, stalk thickening with avid enhancement, and a spectrum of cavernous carotid abnormalities. For each pattern, we emphasize prevalence, typical signal and enhancement characteristics, and reported correlations with biochemical remission or persistence of disease. Finally, we propose a simple flowchart that integrates biochemical status with these reproducible imaging findings to stratify patients into expected postoperative change, probable residual/recurrent adenoma, vascular complication, or indeterminate category. This pattern-based biochemical–radiologic framework is intended to help radiologists and endocrinologists interpret mid-term postoperative pituitary MRI in acromegaly more confidently and consistently.

## 1. Introduction

Acromegaly is an uncommon systemic disorder caused in most cases by autonomous growth hormone (GH) secretion from a somatotroph pituitary neuroendocrine tumor (PitNET), leading to elevated insulin-like growth factor-1 (IGF-1) and multisystem morbidity [1]. Meta-analyses estimate a pooled incidence of approximately 0.38 per 100,000 person-years and a prevalence of about 6 per 100,000 [2]. The World Health Organization (WHO) has updatedthe pathologic classification framework in 2022, which designates these lesions as PitNETs, emphasizes transcription-factor lineages and links histophenotypic features to behavior and outcomes [3]. Radiology plays a major role in the treatment strategy designation from medication regimen selection to surgical planning based on tumor size, anatomical extent, and crucial structure involvement [4]. Even though transsphenoidal surgery (TSS) is the preferred definitive therapy for most patients, biochemical control varies with tumor size, tumor behavior, and experience of the surgeon. Our literature review showed that short- and long-term biochemical control following TSS spans roughly 40–80%, and more interestingly, patients meeting early postoperative biochemical remission criteria, late biochemical recurrence, may occur [5,6].

In this regard, a cohort with long follow-ups showed that biochemical recurrence occurred in 3–4% of population over 7–15 years, which reinforces the importance of standardized radiologic surveillance [7]. Guidelines converge on a coordinated post TSS assessment that pairs biochemical testing with imaging. IGF-1 (with or without GH dynamics) should be reassessed after surgery once early postoperative changes have settled, with many panels recommending the first evaluation at ≥12 weeks; thereafter, IGF-1 is typically monitored every 3–6 months in the first year and then every 6–12 months [8,9]. On the other hand, Magnetic Resonance Imaging (MRI) 3–6 months following TSS is considered as baseline for future comparison and thereafter when biochemistry or clinical status suggest hormonally active disease [10]. The prefect timing of this baseline MRI is controversial since immediate postoperative studies are vulnerable to hemostatic materials, fat grafts, and inflammatory enhancement. Although dynamic contrast-enhanced (DCE) MRI criteria is helpful to separate residual tumor from expected postoperative changes, most institutions still consider the baseline study at least 3 months following TSS [11]. A late postoperative MRI baseline improves specificity for residual disease and provides a stable comparator for future scans [10,12].

This pictorial review focuses on the late postoperative pituitary MRI in acromegaly and emphasizes a practical, pattern-based approach to distinguishing expected postoperative findings from residual or early recurrent tumor, based on the biochemical observations. Our aim is to harmonize radiologic descriptors with endocrine decision points and to provide reproducible imaging cues that can trigger prospective confirmation when management hinges on an imaging call.

## 2. Normal Pituitary and Adenoma MRI Findings

On high-resolution precontrast T1-weighted MRI, the normal anterior pituitary is of intermediate signal intensity, broadly similar to adjacent brain parenchyma and slightly higher than white matter, while the posterior lobe appears as a discrete intrinsically hyperintense focus—the posterior pituitary bright spot—along the dorsal aspect of the gland. Thin-section coronal and sagittal T1WI delineate the anterior lobe within the sella, the midline infundibulum, and the T1-bright posterior lobe, with homogeneous enhancement of both anterior lobe and stalk after gadolinium injection [13,14]. This T1-hyperintense focus is identifiable in essentially all normal glands, with typical craniocaudal dimensions of 2–8 mm, and confirm that it reflects normal neurohypophyseal tissue rather than pathology [14]. On T2-weighted imaging, the anterior lobe demonstrates intermediate signal similar to gray matter, whereas the posterior lobe loses much of its T1 hyperintensity and is less conspicuous, allowing the anterior/posterior interface to be appreciated primarily on T1-weighted sequences [13,15]. Autologous fat grafts exhibit a high T1 signal that can obscure enhancing adenomatous tissue or mimic persistent disease. Therefore, applying fat suppression nulls this background signal to clearly highlight true vascular enhancement during dynamic imaging. The normal anterior pituitary enhances briskly and homogeneously via the hypophyseal portal system, with a characteristic time-to-peak of roughly 80 ± 10 s after contrast injection and subsequent gradual wash-out, yielding a smooth time–signal curve [16,17]. Earlier dynamic MR and CT work similarly demonstrates that the optimal temporal window to maximize contrast between the normally enhancing gland and any focal lesion falls in the early post-contrast phase (approximately 45–60 s after injection), as normal tissue enhances strongly and uniformly in this interval [18,19,20].

In acromegaly, the overwhelming majority (95%) of cases are caused by a growth hormone (GH)-secreting pituitary adenoma, and the resulting chronic GH excess drives hepatic overproduction of IGF-1 [21,22]. GH-producing somatotrophs are the most abundant anterior pituitary cell population (roughly half of all adenohypophyseal cells) and are concentrated predominantly in the lateral wings of the gland [23,24,25]. On imaging, these tumors therefore most commonly appear as off-midline nodules originating from the lateral aspect of the anterior lobe and expanding toward one cavernous sinus; as they grow, they frequently present as macroadenomas with suprasellar extension and varying degrees of cavernous sinus invasion at the time of acromegaly diagnosis [13,21,22,23].

On conventional MRI, pituitary adenomas including somatotroph lesions are typically well-circumscribed masses within the anterior lobe that are iso- to mildly hypointense relative to normal pituitary tissue on T1-weighted images, with variable T2-weighted signal that ranges from hypo- to hyperintense compared with gray matter, and they enhance after gadolinium but often more slowly and heterogeneously than the surrounding gland [13,15,19]. Functional microadenomas characteristically exhibit delayed and less intense enhancement: time-to-peak for microadenomas averages around 90 s, compared with approximately 80 s for the normal anterior lobe, and several DCE studies have demonstrated that this difference keeps the time–intensity curves separable between roughly 60 and 140 s, a window exploited by targeted pituitary DCE protocols [16,17,20,26]. In growth hormone-secreting adenomas specifically, T2-weighted signal carries additional biologic information: T2 signal intensity (classified visually or quantified as a T2 ratio) correlates with histologic granulation pattern, baseline GH/IGF-1 levels, and response to somatostatin analogs, densely granulated somatotroph adenomas tending to be T2-hypointense, and sparsely granulated, often larger and more invasive tumors tending to be T2-hyperintense [22,27,28,29]. Together, these preoperative MRI characteristics, the lateral intra-glandular origin of somatotroph adenomas, their frequent macroadenoma presentation with extrasellar extension, their relatively delayed and hypo-intense dynamic enhancement, and their subtype-specific T2 signal, provide the baseline framework against which the postoperative patterns described in Section 3 are interpreted.

## 3. MRI Findings1

The MRI protocol is discussed thoroughly in Table 1. This enables us to discriminate expected post TSS content from potential residual/recurrent disease [13]. We designed a practical imaging approach to read pituitary MRI of patients with rising biochemical markers. These correlations were validated with at least two more follow-up imaging (C1–C8).

### 3.1. Presence of Packing Materials

**Figure 1:**
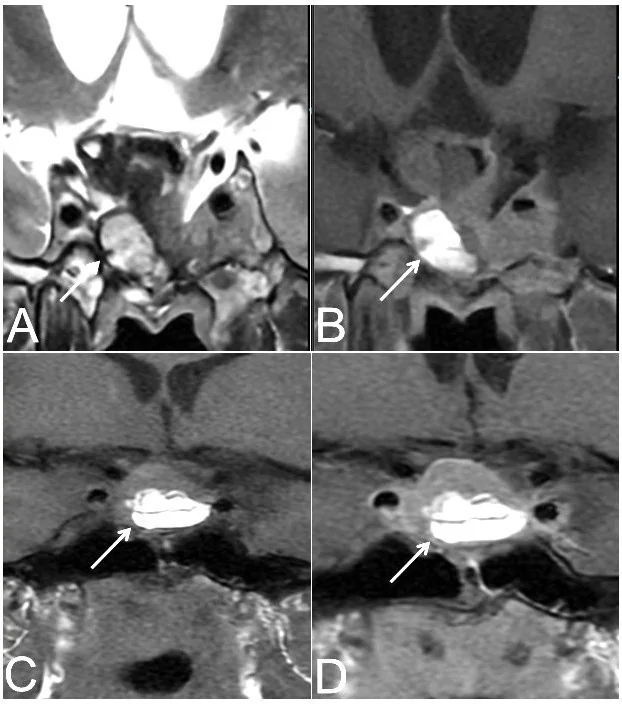
Expected Post-surgical MRI Findings. Surgical packing materials after transsphenoidal surgery. 3 months post transsphenoidal surgery MRI is shown on coronal T2WI (**A**) and unenhanced coronal T1WI (**B**). The images demonstrate a typical postoperative fat flap (arrow) along the right side of the sella turcica, corresponding to a serum IGF-1 level of 15 ng/mL (within normal range) consistent with expected postoperative imaging observations. Another patient with acromegaly, status post transsphenoidal surgery, is shown on unenhanced coronal T1WI (**C**) and contrast-enhanced coronal T1WI (**D**). The images demonstrate a similar gelfoam/fat flap (arrow) within the sella turcica.

Autologous fat and hemostatic sponges are routinely placed along the sellar floor and the base of the resection cavity to obliterate dead space and reduce the risk of CSF leakage. Correct identification of these benign materials prevents mislabeling postoperative change as residual tumor and avoids unnecessary adjuvant therapy; classic and contemporary pituitary MRI series were developed specifically to codify these signatures and standardize postoperative interpretation [30,31]. 3–6 months follwing transsphenoidal surgery, the low-signal material along the right side of the sella on coronal T2WI and the corresponding T1-hyperintense, non-enhancing fat flap, represent typical postoperative packing rather than residual adenoma (Figure 1). In our example, the first patient’s normalized IGF-1 (15 ng/mL) and stable, sharply marginated, T1-bright, non-enhancing tissue along the sellar wall are characteristic of autologous fat, while the second patient shows a similar gelfoam/fat composite within the sella on unenhanced and contrast-enhanced T1WI without any new nodular enhancement. At this 3-month time point, such non-enhancing, stable packing material—particularly when IGF-1 has normalized—should be interpreted as expected postoperative change rather than residual GH-secreting adenoma.

Autologous fat and hemostatic sponges are routinely placed along the sellar floor and resection cavity to obliterate dead space and reduce CSF leak risk. Correctly recognizing these materials prevents labeling expected postoperative change as residual tumor and avoids inappropriate adjuvant therapy; classic pituitary MRI series were designed to codify these appearances [15,16]. At 3–6 months after transsphenoidal surgery, a stable T1-hyperintense, non-enhancing fat flap along the sellar wall or floor, as in Figure 1, especially in a patient with normalized IGF-1, almost always represents autologous fat or a fat/Gelfoam composite rather than residual adenoma.

Several imaging series describe the mid-term MRI behavior of sellar packing. In a large cohort of 154 patients (including 59 with acromegaly), Bladowska et al. showed that intrasellar autologous fat remains T1-hyperintense, relatively T2-intermediate, and non-enhancing for many months to years, with only gradual volume loss; hemostatic and cellulose materials were usually no longer visible by about 1 month [30]. Dina et al. followed 10 patients with serial MRI and found that by 4–9 months, Gelfoam packing had completely or nearly completely resorbed in 8/10 cases, whereas fat-packed defects persisted as T1-bright foci, leaving a smaller but often deformed gland. In a prospective study including 10 acromegaly patients, Ghorbani et al. obtained MRIs at 48 h, 2 weeks, and 3 months after surgery (fat pad + Surgicel + gelfoam reconstruction) and reported that at 3 months, packing material remained visible in all patients, with a hyperintense or mixed T1 signal in 82% and evolving T2 signal from hyperintense to hypointense over time, yet residual tumor could still be confidently delineated when present [32]. More long-term skull-base series confirm that fat flaps shrink rather than disappear abruptly: Cossu et al. measured fat grafts in 72 pituitary patients and found that about half of the graft volume is lost between 3 months and 1 year, with T1 signal gradually decreasing as the graft fibroses, but remaining distinct from tumor [33]. Together with descriptive reviews noting that fat appears T1-bright and non-enhancing while Gelfoam is variable but often isointense with a hypointense center, both showing progressive atrophy [31], these data support the interpretation of a stable, non-enhancing T1-bright fat flap at 3–6 months as benign packing, especially in a biochemically cured patient.

However, several studies highlight situations where packing can complicate interpretation or even mimic residual tumor. Steiner et al. reported that implanted gelatin foam may appear as a circularly enhancing endosellar mass early on, and in one of 25 macroadenoma patients they could not reliably distinguish residual tumor from implanted material on postoperative MRI, despite using preoperative appearance as a guide [31]. Ghorbani et al. noted that identical packing material (fat + Surgicel + gelfoam) exhibited heterogeneous signal characteristics between patients, likely due to variable blood staining, raising the possibility of confusing blood-impregnated packing with tumor on isolated scans [32]. Kılıç et al., in a prospective series of 80 adenomas imaged at 24 h and then 3, 6, 9, and ≥12 months, emphasized that fat takes longer to resorb than Gelfoam but stressed that blood and early postoperative changes may still obscure tumor margins; they therefore relied heavily on the very early (24 h) study as the reference for later scans [34]. More recent skull-base reconstruction papers also argue that bulky fat packing can interfere with postoperative sellar assessment and have explored collagen-based or mucosal graft repairs to minimize imaging artifacts [35,36]. These observations remind us that packing is not always invisible diagnostically and that enhancing or heterogeneous components within the surgical bed cannot be dismissed solely on the basis of prior fat or gelfoam use.

In practical terms, for 3–6-month postoperative MRI in acromegaly, the radiologist should first recognize the expected signature of packing: a T1-hyperintense, non-enhancing fat flap or composite fat/gelfoam plug along the sellar wall or floor, often slightly smaller than on immediate postoperative imaging but still clearly present. When this appearance is stable compared with earlier scans and accompanied by biochemical remission (normalized IGF-1 and appropriate GH nadir), it can be safely regarded as benign postoperative change rather than residual adenoma, as illustrated in Figure 1. Conversely, new or enlarging enhancing nodules within or adjacent to the packing, progressive mass effect, or unexpected change in packing signal, especially in the setting of rising IGF-1, should prompt suspicion for residual or recurrent GH-secreting tumor and trigger closer follow-up, dynamic contrast-enhanced MRI, and/or surgical or medical re-evaluation.

### 3.2. Late-Phase Enhancing Lesion

**Figure 2:**
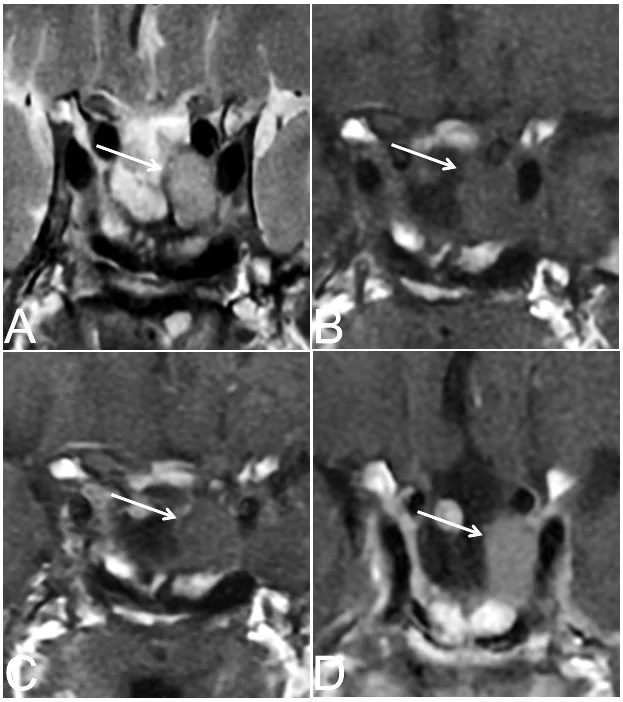
Recurrent Disease. 4 months post transsphenoidal surgery, camewith rising serum IGF-1 (110 ng/mL,previously20 ng/mL). MRI demonstrates a well-defined lesion (arrow) along the left margin of the sella turcica. The lesion appears mildly hyperintense on coronal T2WI (**A**) and hypointense on coronal T1WI (**B**). On dynamic assessment, no contrast uptakeis appreciated on the early phase (**C**), whilediffuse mild homogeneous enhancement demonstarted on the delay phase (**D**), consistent with recurrent disease.

At 3–6 months after transsphenoidal surgery, a well-defined soft-tissue nodule in the operative bed that is T2 mildly hyperintense, T1 hypointense, and shows delayed mild homogeneous enhancement (Figure 2) should be viewed with a high index of suspicion for residual or recurrent adenoma, especially in a patient with rising IGF-1. In the example case, the patient has a new focal lesion along the left sellar margin at 4 months, with no appreciable early dynamic enhancement but progressive, homogeneous enhancement on delayed images, in parallel with IGF-1 rising from 20 to 110 ng/mL. This pattern differs from thin peripheral or rim-like enhancement of postoperative change and from the non-enhancing, T1-bright packing shown in C1: here, the lesion is parenchymal, nodular, and follows adenoma-like contrast kinetics. Current neurosurgical guidelines recommend the first routine postoperative MRI at about 3–4 months specifically to assess for residual tumor, and at that time, a discrete enhancing nodule in the resection cavity, particularly with biochemical relapse, is usually interpreted as persistent disease rather than simple scar or packing [37,38].

Several MRI studies support the idea that nodular or thick enhancing tissue in the surgical bed correlates strongly with residual adenoma. Kim et al. used immediate postoperative dynamic contrast-enhanced sella MRI in pituitary adenomas and found that enhancing tissue thicker than 3.9 mm with a nodular pattern was highly predictive of residual tumor, with a reported sensitivity of 89%, specificity of 97% and accuracy of 94% for that cut-off; thinner, flat enhancement was usually postoperative change [11]. A large contemporary imaging review summarizing these data notes that dynamic MRI is particularly useful postoperatively: residual tumors tend to show a nodular contrast area, whereas pure postoperative change typically enhances only at the margins without a solid nodule [37]. Hassan et al. prospectively studied 30 patients with non-functioning macroadenomas using early MRI and diffusion-weighted imaging and then re-imaged them at 6 months; 13 patients ultimately had residual adenoma and 17 had only granulation tissue. Early contrast-enhanced MRI alone (evaluating patterns such as peripheral vs. nodular enhancement) achieved a sensitivity of 84.6% and specificity of 94.1% for residual tumor when compared with the 6-month reference standard, and nodular enhancement that persisted or became more conspicuous at 6 months correlated with residual adenoma rather than postoperative granulation [39]. A classic early postoperative dynamic study by Yoon et al. similarly shows that dynamic MR is very effective in differentiating residual tumor from postoperative changes, with adenoma enhancing as a compact focus distinct from the thin, inhomogeneous enhancement of the surgical bed; though these data are from the first postoperative week, the same morphology (persistent, nodular enhancing tissue) tends to be the pattern that remains at 3–6 months [40]. Together, these studies support using a small, well-defined enhancing nodule as a radiologic marker of residual or recurrent adenoma, especially when IGF-1 is rising.

At the same time, not every mildly enhancing lesion at 3–6 months represents hormonally active tumor, and MRI alone has limited specificity for endocrine status. In the Hassan series, more than half of patients had enhancing postoperative granulation tissue rather than adenoma, and some enhancing masses regressed or lost enhancement by 6 months, emphasizing that early enhancement patterns can reflect healing tissue rather than true tumor [39]. Skull-base reviews similarly warn that enhancing granulation tissue in the resection cavity can mimic residual tumor; granulation typically involutes on serial imaging, whereas true adenoma persists or grows [11,41,42]. Post-surgical pituitary series have also shown that the MRI appearance of the postoperative gland does not necessarily parallel hormonal function, with significant discordance between imaging impression and endocrine status [30,43]. In acromegaly, this disconnect cuts both ways: some patients show biochemical remission despite small residual tumor masses on imaging, and conversely, others develop biochemical recurrence without clear regrowth on serial MRI [44,45]. Case reports using PET/MRI even demonstrate apparent residual tumor on conventional MRI that proves metabolically inactive and nonfunctioning [46]. These data underline that a mildly enhancing nodule at 3–6 months cannot be equated with active disease in isolation; its significance depends on dynamics over time and on hormone levels.

In practice, at 3–6 months after transsphenoidal surgery for acromegaly, a new or enlarging, well-defined, mildly enhancing nodule in the operative bed—particularly one ≥3–4 mm thick, with nodular rather than thin marginal enhancement and delayed homogeneous uptake—should be considered highly suspicious for residual or recurrent adenoma when accompanied by rising IGF-1 or GH, as in Figure 2. Dynamic MRI and, where available, adjunct techniques such as DWI or high-resolution thin-slice imaging can help distinguish compact tumor from more diffuse granulation tissue, but none of these substitute for biochemical follow-up [39,47]. Small, stable enhancing nodules in a patient with durable biochemical remission are more likely to represent scar or nonfunctioning residue and can usually be monitored with interval MRI rather than treated immediately as recurrence. The key clinical takeaway is that at the 3–6-month scan, mild enhancing lesions in the surgical bed should trigger careful correlation with IGF-1/GH trends and prior imaging; growth and biochemical relapse favor recurrent adenoma, whereas stability or regression and normal hormones favor benign postoperative change.

### 3.3. Surgical Bed Thin Rim Enhancement

At 3–6 months after transsphenoidal surgery, a cystic surgical-bed cavity that is low-signal on T1WI, fluid-like on T2WI, and shows a smooth, thin peripheral rim with fine internal septal enhancement on post-contrast T1WI without any solid nodular component most often represents postoperative healing of the resection cavity rather than recurrent adenoma. By about four to nine months, absorbable hemostatic materials typically resorb, the sellar contents decrease in height, and the normal gland re-expands; accordingly, a thin peripheral rim of enhancement usually stabilizes or regresses during this interval [38,48,49]. For isntance inFigure 3, 3 months after surgery for acromegaly has exactly this appearance, together with repeatedly normal IGF-1 levels and no new symptoms. In this context, the rim and septal enhancement are best interpreted as granulation tissue lining a postoperative fluid cavity or pseudocyst in the resection bed, representing expected postoperative change rather than residual GH-secreting tumor.

Early dynamic MRI work by Yoon et al. (83 surgically proven adenomas, including 22 GH-secreting tumors) showed that patients could be grouped by enhancement pattern in the postoperative mass: no enhancement, peripheral rim enhancement, nodular enhancement, or combined nodular and rim. Importantly, all 22 residual tumors occurred in the nodular or combined groups, whereas none of the 13 patients with pure peripheral rim enhancement had residual adenoma on long-term hormonal and MRI follow-up (6–48 months) [40]. A later dynamic MRI study by Kim et al. summarized Yoon’s data and emphasized that peripheral rim enhancement typically disappeared on follow-up as the normal gland re-expanded into the fossa, again supporting its benign, postoperative nature [11]. In Hassan et al.’s study, early peripheral rim enhancement was seen in 7/30 patients; by 6 months, three still had a linear/rim pattern, two had no enhancement, and two had converted to nodular enhancing masses with clinical deterioration and were classified as residual tumor [39]. In this study, using enhancement pattern plus ADC values, early MRI alone achieved an NPV of 89% and early DWI an NPV of 93% for excluding residual tumor, reinforcing that thin, non-nodular rim enhancement is usually granulation tissue rather than adenoma [39]. Finally, a sellar reconstruction series from Ismail et al. showed that hemostatic packing often develops a peripheral rim of enhancement caused by granulation tissue beginning around 3 months post-op and lasting up to 8 months, exactly overlapping our 3–6-month surveillance window [50]. However, rim enhancement must still be interpreted cautiously. In Hassan et al., two of seven patients with early peripheral rim enhancement ultimately proved to have residual adenoma at 6 months, when the rim evolved into persistent nodular enhancement and clinical status worsened [39]. Rim enhancement alone is not perfectly specific, and evolution over time, wall thickness, presence of any nodular component, diffusion characteristics, and the clinical and biochemical picture must all be considered.

In practice, for a 3–6-month postoperative MRI in acromegaly, a smooth, thin peripheral rim of enhancement with delicate internal septa around a cystic surgical-bed cavity, which is stable or decreasing in size and occurs in a patient with normal, stable IGF-1, is most consistent with postoperative granulation tissue and healing of the resection cavity or packing material, and carries a high negative predictive value for residual adenoma. Early dynamic MRI and follow-up data show that residual or recurrent tumor is much more likely when enhancement becomes thick or nodular or when a solid enhancing component appears or enlarges, especially in parallel with rising IGF-1/GH [39,40]. Thus, thin rim enhancement should generally be reported as an expected postoperative finding, while new or progressive thickening of the rim, development of mural nodules, restricted diffusion, or biochemical relapse should prompt closer surveillance, repeat dynamic MRI, and consideration of further intervention.

### 3.4. Cavernous Sinus Remaining/Recurrent Adenomatous Tissue

**Figure 4:**
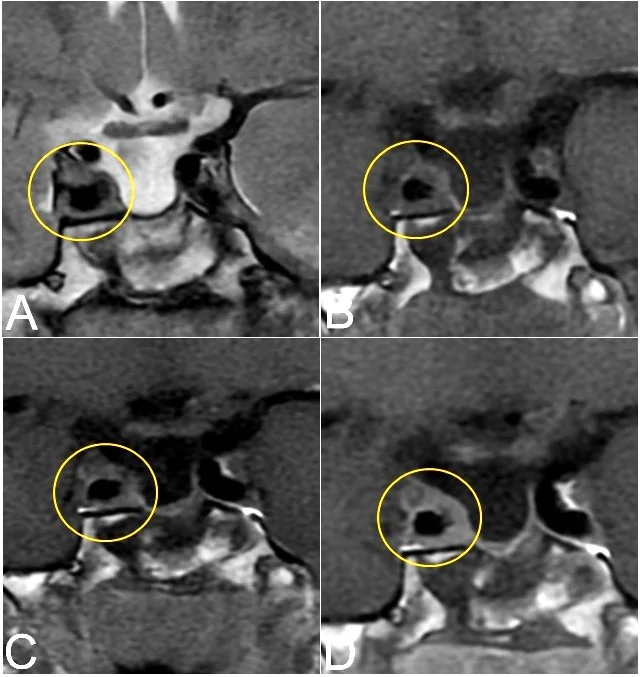
Cavernous Sinus Recurrent Adenomatous disease. Patient presented with rising serum IGF-1 (180 ng/mL, previously 96 ng/mL) and was referred for MRI (5 months since surgery). Coronal T2WI (**A**) and coronal T1WI (**B**) demonstrateabnormal signal (Circle) around the right cavernous carotid artery and within the right cavernous sinus, appearing hypointense on T2WI and isointense on T1WI relative to the white matter. On dynamic imaging, minimal heterogenous contrast uptake is seen in the early phase (**C**), with diffuse mild enhancement on the delayed phase (**D**). These observationsrepresent either residual or recurrent disease, which needs comparsion to the preoperative MRI.

Cavernous sinus residual or recurrent adenomatous tissue at 3–6 months typically appears as asymmetric soft tissue encasing or filling part of the cavernous sinus around the internal carotid artery, isointense to brain on T1, often T2 hypointense, and showing mild, relatively delayed enhancement (for example see Figure 4). GH-producing PitNETs are frequently T2-hypointense and can enhance less avidly than other adenoma subtypes on dynamic MRI, so a subtly enhancing, T2-dark plaque hugging the cavernous carotid in a patient with rising IGF-1 is highly suspicious for persistent somatotroph tumor rather than postoperative change [37]. In modern series, gross total resection is usually defined by the absence of any visible adenomatous tissue on MRI obtained about 3–6 months after surgery, reinforcing that any residual soft tissue in the cavernous sinus at this time point should be assumed to represent tumor until proven otherwise [51]. Thus, in a 5-month postoperative acromegaly patient with rising IGF-1, infiltrative cavernous sinus tissue with these signal and enhancement features is best interpreted as residual or recurrent hormonally active disease.

Multiple studies link cavernous sinus involvement to incomplete resection and poorer biochemical control in acromegaly. Large mixed-adenoma cohorts show that radiological and surgical invasiveness (largely driven by cavernous sinus extension on Knosp grading) strongly predict lower rates of gross total resection and shorter progression-free survival; in one 903-patient series, radiological invasiveness carried a >5-fold higher hazard of recurrence/progression, with GTR defined on 3–6-month postoperative MRI [51]. In GH-secreting tumors, a meta-analysis of retrospective acromegaly series using 3-month MRI to assess extent of resection found that higher Knosp grades (3–4), which correspond to probable carotid encasement and cavernous sinus extension, are associated with lower odds of early biochemical remission and a greater need for adjuvant radiotherapy or medical therapy [52]. In a meta-analysis, remission rates were approximately 48% for invasive macroadenomas compared with over 70% for non-invasive lesions, underscoring the prognostic weight of cavernous sinus disease [53]. Contemporary acromegaly guidelines and surgical reviews likewise highlight cavernous sinus invasion as an independent predictor of failure to achieve biochemical remission, second only to very high preoperative GH levels, and recommend a high index of suspicion for persistent or recurrent disease when postoperative imaging shows residual cavernous sinus tissue [54]. In more focused GH-adenoma series where surgeons aggressively explore the cavernous sinus, endocrinologic remission still drops dramatically as invasion progresses from medial to carotid-encasing compartments, illustrating that residual cavernous tissue is both common and clinically relevant [55].

At the same time, radiologic assessment of cavernous sinus invasion is imperfect and can overcall tumor where only compression or venous enhancement exists. A recent series comparing preoperative MRI reports to intraoperative findings found that although 70% of patients were reported to have cavernous sinus invasion, only 9% actually had invasion confirmed at surgery (PPV 9.1%, NPV 100%), emphasizing that radiographic suspicion alone has low specificity [56]. Broader radiologic review of aggressive pituitary tumors stresses that distinguishing true invasion from mere lateral extension or venous congestion is often challenging; invasion is considered radiologically certain mainly when there is complete encasement of the intracavernous carotid and disruption of normal venous compartments [55]. A large classification study also documents that Knosp-based radiological grading yields a substantial rate of false-positive invasive labels, prompting recommendations to combine MRI with surgical and histological impressions rather than relying on imaging alone [51]. These data imply that subtle or equivocal cavernous sinus enhancement on a mid-term postoperative MRI particularly without clear arterial encasement or a discrete nodular component may not always equate to active tumor and must be interpreted alongside endocrine status.

In practice, new or persistent T2-hypointense, T1-isointense soft tissue encasing or filling the cavernous sinus at 3–6 months, showing mild, fairly homogeneous delayed enhancement, is high-risk for residual GH-secreting adenoma when IGF-1 is incompletely controlled or rising, as in Figure 4, and should prompt explicit reporting and multidisciplinary discussion about re-operation, radiosurgery, or medical escalation. Conversely, small, stable areas of more linear or thin soft tissue along the cavernous sinus wall, without progressive thickening, mass effect, or clear carotid encasement, and in the setting of normalized IGF-1, may represent postoperative scarring or venous changes rather than true residual tumor and can often be monitored with serial MRI. Integrating preoperative Knosp grade, intraoperative documentation of cavernous sinus exploration, and the trajectory of IGF-1 and GH at the 3–6-month visit is essential: cavernous sinus soft tissue in a biochemically uncontrolled acromegaly patient should be presumed residual disease until proven otherwise, whereas similar tissue in a biochemically cured patient warrants careful follow-up rather than automatic labeling as recurrence.

### 3.5. Stalk Deviation

**Figure 5:**
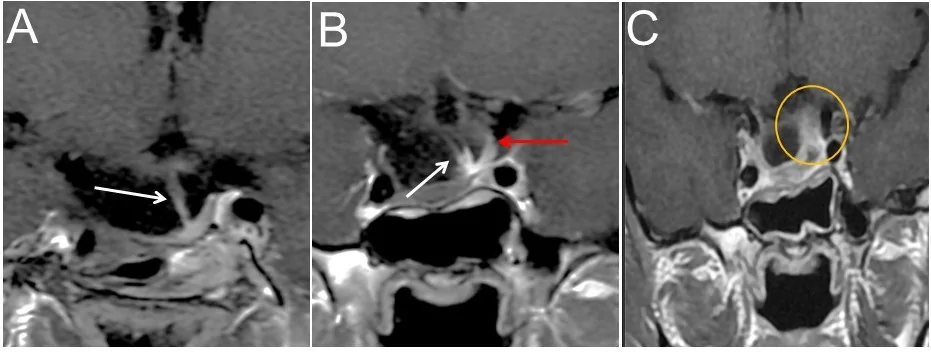
Pituitary Stalk Observations Coronal CE-T1WI (**A**) in a postoperative patient demonstrates leftward deviation of the pituitary stalk withnormal thickness (arrow), correlating with normal biochemical and clinical findings, and representing a normal postoperative observation. Coronal CE-T1WI (**B**) shows a thickened pituitary stalk (whitearrow) with an associated enhancing suprasellar band (redarrow), suggesting hypervascular recurrent disease in the context of rising IGF-1 levels and recurrent clinical symptoms. Coronal CE-T1WI (**C**) obtained 5 months after surgery demonstrates a severely thickened pituitary stalk with deviation (circle), concerning for active disease, given the rising elevated IGF-1 level of 90 ng/mL.

At 3–6 months after transsphenoidal surgery, a deviated but slender pituitary stalk on coronal contrast-enhanced T1WI like in Figure 5 usually reflects postoperative gland shift or the effect of packing rather than recurrent tumor. High-resolution MRI studies show the normal stalk is thin (roughly 2–3 mm in diameter, tapering from 3.2 mm at the optic chiasm to 2 mm at the pituitary insertion) and enhances homogeneously [57,58]. Postoperative series demonstrate that, after adenoma resection, the stalk often changes position and length as the diaphragm and gland descend and packing material resorbs, with a tendency to recenter over the first few months [58]. In contrast, the appearances in Figure 5, diffuse stalk thickening with intense enhancement and an associated enhancing suprasellar band are more in keeping with hypervascular pathological tissue. In a patient with acromegaly and rising IGF-1 at 3–6 months, such thickened, enhancing stalk tissue is highly suspicious for residual or recurrent adenoma (or other infundibular pathology) rather than simple postoperative displacement.

Several lines of evidence support this distinction. In a 108-patient series, Zhang et al. measured stalk morphology pre-, early post-, and mid-term (3–4 months) after transsphenoidal resection: preoperative deviation was common, but by mid-term follow-up about half of deviated stalks had recentered, and the stalk generally appeared stretched toward a normal configuration as gelfoam and fluid within the cavity were resorbed [58]. Early postoperative work by Yoon et al. (≤1 week) similarly described transient stalk thickening and deviation as part of normal postoperative change rather than tumor [40]. In intraoperative MRI, Becker et al. identified a minimal stalk diameter ≥ 2.9 mm as a strong predictor of new adrenocortical insufficiency (OR ~ 29) and AVP deficiency (OR ~ 6), yet by the 3-month follow-up the stalk diameter no longer differed between patients with and without these deficits, suggesting that acute swelling typically regresses by the mid-term interval [59]. Beyond the postoperative setting, large series of pituitary stalk lesions confirm that true pathological thickening is usually more substantial: Kluczyński et al. reported a mean stalk diameter of 6.1 mm (range 2.3–15 mm) in 44 patients with inflammatory or neoplastic stalk disease, with neoplastic lesions (including somatotroph adenomas and a stalk-based recurrence of Cushing disease) showing marked thickening and homogeneous enhancement and frequently associated with pituitary dysfunction [60]. Similarly, Ling et al. found that in a 325-patient cohort with stalk thickening, a width of 4.75 mm predicted central diabetes insipidus plus anterior pituitary deficiency with moderate sensitivity (69%) and specificity (71%), underscoring that clearly thickened stalks are usually abnormal [61]. Taken together, these data support the view that a markedly thickened, avidly enhancing stalk at 3–6 months, especially if contiguous with enhancing suprasellar tissue and accompanied by rising IGF-1, is much more likely to represent active disease than normal postoperative remodeling.

However, stalk deviation and thickening are not specific for recurrent adenoma and must be interpreted with caution. Deviation angle itself primarily reflects mechanical stretching and diaphragm movement: Xue et al. and Lin et al. showed that larger changes in stalk deviation angle and greater diaphragma sellae sinking after surgery were independent predictors of early diabetes insipidus and delayed hyponatremia, respectively, highlighting vulnerability of the hypothalamo-hypophyseal tract but not directly indicating tumor persistence [62,63]. Broad stalk-lesion series in adults and mixed cohorts also demonstrate that many thickened stalks are inflammatory (e.g., lymphocytic hypophysitis, neuroinfundibulitis) or histiocytic rather than neoplastic: in Kluczyński’s 60-patient cohort, 25% of lesions were inflammatory and often showed subsequent reduction or stabilization of stalk size on serial MRI [60], while Devuyst et al. found that among 38 adults with central DI and stalk thickening, only 29% had neoplastic lesions (germinoma, metastasis, etc.), with the remainder due to inflammatory or infiltrative causes [64]. Hâna’s review of pituitary stalk enlargement in adults similarly stresses that isolated stalk thickening has a wide differential; in many cases a watch-and-wait strategy with repeat MRI at 3–6 months is recommended, because some lesions regress (e.g., hypophysitis) and others only declare themselves over time [57]. Moreover, in these series, IGF-1 is often low or normal, emphasizing that a thick stalk does not automatically equate to somatotroph adenoma recurrence.

In practical terms, a deviated but slender, smoothly enhancing stalk at 3–6 months in a biochemically cured acromegaly patient, should be regarded as a common, largely benign postoperative finding that reflects gland and stalk repositioning rather than recurrent disease. By contrast, new or progressive stalk thickening above roughly 3 mm with strong, diffuse enhancement, particularly when forming an enhancing suprasellar band or nodular tissue contiguous with the surgical bed (Figure 5), should raise concern for residual or recurrent adenoma or another stalk lesion when accompanied by rising IGF-1 or new pituitary dysfunction. Given the overlap with inflammatory and other non-adenomatous etiologies, such imaging must be integrated with the full clinical context: prior tumor type and invasiveness, radiation history, systemic signs of inflammation or malignancy, and detailed endocrine testing. For the radiologist reviewing 3–6-month postoperative MRI in acromegaly, the key takeaway is to reassure when the stalk is merely off-midline but thin and stable, and to flag a thickened, intensely enhancing stalk or enhancing suprasellar band, especially in the setting of biochemical relapse, as a high-risk pattern that warrants closer multidisciplinary evaluation and, in many cases, further treatment.

### 3.6. Granulation Tissue

**Figure 6:**
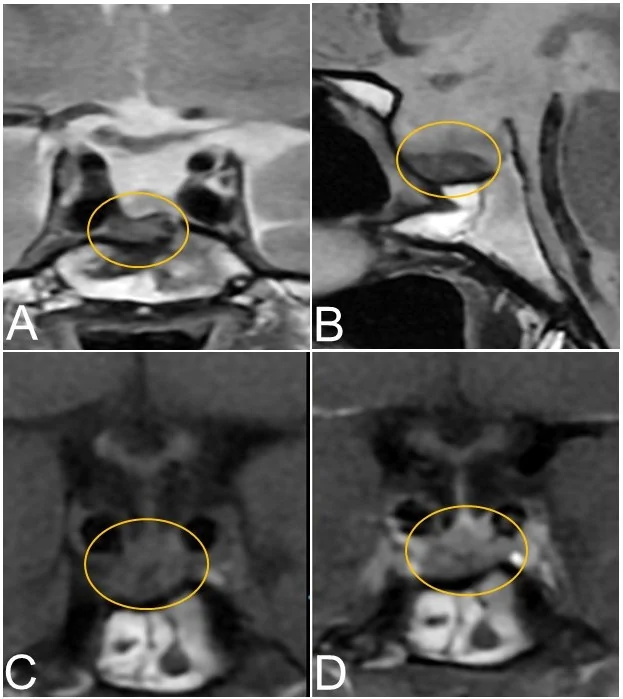
Granulation Tissue MRI Features. Imaging surveillance, 3 months after TSS, I. Clinical and biochemical findings, including IGF-1 levels, were within the normal ranges. Coronal and sagittal T2WI (**A**,**B**) show low-signal tissue (circle) in the floor of the sella turcica. On coronal pre-contrast T1WI (**C**), the lesion appears heterogeneous and predominantly isointense, with heterogeneous contrast enhancement on coronal post-contrast T1WI (**D**). In the context of normal biochemical study and clinical exam, these MR findings are consistent with granulation tissue.

Granulation tissue in the postoperative sella typically appears, as in Figure 6, as low signal relative to white matter on T2-weighted images, with heterogeneous but predominantly isointense signal on T1 and patchy or heterogeneous enhancement along the sellar floor at 3–6 months after surgery. In a patient 6 months post-transsphenoidal surgery for acromegaly with normal IGF-1, this sheet-like, floor-based tissue that does not form a discrete expansile nodule is most consistent with postoperative granulation tissue and scar rather than residual adenoma. This interpretation leans heavily on the combination of imaging pattern (ill-defined, heterogeneous, low-T2 tissue along the operative plane), stability or slight regression over time, and concordant biochemical remission.

Several series support this reading of postoperative enhancing tissue as benign granulation when the pattern is thin or sheet-like and the patient is biochemically controlled. Yoon’s early postoperative dynamic MRI study of pituitary adenomas showed that thin or irregular peripheral enhancement around the surgical bed often regressed over 4–9 months with re-expansion of normal gland and normalized hormonal tests [40]; Steiner had already suggested that such peripheral enhancement seen several months after surgery likely reflects granulation tissue at the resection margin [31]. More recently, Hassan et al. followed 30 non-functioning macroadenomas; 17/30 lesions proved to be postoperative granulation tissue at 6 months and showed higher ADC values (1.3–1.5 × 10^−3^ mm^2^/s) than residual adenoma (0.83–0.86 × 10^−3^ mm^2^/s) [39]. Volumetric series likewise treat enhancing but non-nodular postoperative tissue as expected healing: in a 94-patient macroadenoma cohort, Chuang et al. explicitly measured only gland plus granulation tissue in gross total resection cases without radiologic tumor remnant, underscoring how common such tissue is in successful resections [65]. However, contemporary PitNET imaging guidelines recommend interpreting postoperative enhancement in conjunction with serial imaging and endocrine data rather than a single morphologic criterion, explicitly noting that postoperative scar and granulation may mimic tumor and that advanced techniques such as DWI are adjunctive rather than definitive [32,66].

In practice, heterogeneously enhancing, low-T2 tissue along the sellar floor at 3–6 months without a discrete nodular component, without progressive enlargement, and in the setting of normal or improving IGF-1 should be regarded as granulation tissue and expected postoperative healing rather than immediate evidence of recurrent adenoma. Conversely, the development over time of a new or enlarging nodular focus within this background tissue, especially if its enhancement pattern reproduces the preoperative adenoma or if IGF-1 begins to rise, should raise concern for residual or recurrent tumor and prompt closer surveillance or intervention. Thus, for the 3–6-month MRI, the key is to integrate pattern (sheet-like vs. nodular), signal (low-T2 fibrous tissue vs. adenoma-like), and temporal evolution with biochemical status: most stable, heterogeneously enhancing granulation tissue in a biochemically cured acromegaly patient can be safely monitored, whereas any nodular, enlarging, or discordant component warrants suspicion.

### 3.7. Reappearance of the Posterior Pituitary Bright Spot

**Figure 7:**
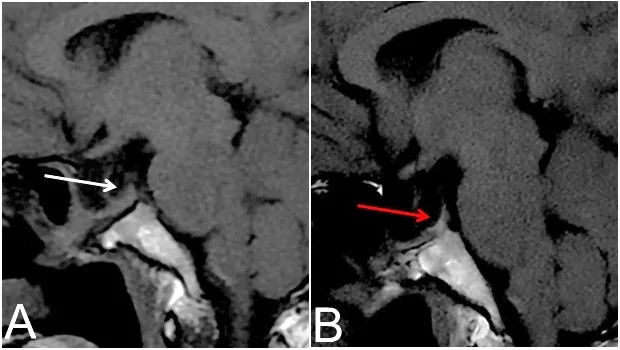
Posterior Pituitary Bright Spot Revisualization(PPBS). Status post transsphenoidal surgery, with a normal postoperative biochemical profile and no clinical signs of recurrence. On sagittal unenhanced T1WI obtained 3 months after surgery (**A**), the PPBS is not visible (white arrow). On follow-up sagittal T1WI performed 6 months after surgery (**B**), the PPBS reappears (red arrow).

On unenhanced sagittal T1-weighted MRI, the posterior pituitary bright spot (PPBS) is the intrinsic T1 hyperintensity of the neurohypophysis, reflecting stored vasopressin/neurophysin granules [13]. In Figure 7, the PPBS is poorly visualized at 3 months but clearly reappears at 6 months in a man who remains clinically well and biochemically cured of acromegaly. Longitudinal postoperative series show that after transsphenoidal surgery, the PPBS can transiently disappear, move, or change in size as mass effect resolves and the posterior lobe descends, before stabilizing at the sellar base by a few months [58,67]. Taken together with normal sodium balance and normal IGF-1, reappearance or persistence of the PPBS at 3–6 months is best interpreted as a supportive sign of preserved or recovered posterior pituitary function, whereas its transient absence is not, by itself, a marker of diabetes insipidus (DI) or poor outcome.

In the general population, the PPBS is visible in the vast majority of adults: in a 1017-subject study, it was present in 95.9% of scans, with absence in about 4%, slightly more often in older patients and in men [68]. Radiologic and endocrine work indicates that the bright spot reflects vasopressin storage; in idiopathic or familial central DI, the PPBS is typically absent while unaffected relatives and controls retain a normal bright spot [13,69]. In pituitary adenoma surgery cohorts, preoperative or early postoperative PPBS is associated with a lower risk of postoperative DI: Wang et al. reported that among 65 adenoma patients, 10/32 with DI versus 1/33 without DI had a negative preoperative PPBS, and logistic regression identified negative PPBS as an independent predictor of postoperative DI [70]. A larger TSS series by Zhang et al. found PPBS present preoperatively in 53.7% of adenoma patients, with 53 patients again showing a PPBS at mid-term (4 months) follow-up despite early postoperative variability; early negative PPBS was associated with a higher crude DI rate, but many patients without DI also had absent PPBS, and most patients ultimately showed a persistent or reappearing bright spot [58]. Saeki’s longitudinal MRI study of large adenomas with ectopic PPBS similarly demonstrated that in all 15 patients, the bright spot remained visible at early (<1 week), intermediate (1–2 months), and late (≥6 months) postoperative scans, typically descending along the stalk as mass effect resolved, and none developed permanent DI [67]. These data support the notion that a stable or reappearing PPBS at 3–6 months, in a patient without polyuria or hypernatremia, is reassuring with respect to neurohypophyseal integrity.

Conversely, multiple studies highlight that the PPBS is an imperfect surrogate for posterior pituitary function. Classic population work by Brooks and later by Klyn et al. showed that a small proportion of otherwise normal subjects have no visible PPBS, indicating that absence can be a normal variant [68,71]. In Wang’s adenoma series, most transient DI cases still had a visible preoperative PPBS (22/32), and one patient without DI had a negative PPBS, underscoring that presence does not exclude, and absence does not guarantee, DI [70]. Zhang et al. further observed that some patients with negative PPBS in the early postoperative period did not develop DI, and concluded that early PPBS loss may reflect dynamic vasopressin release or technical factors rather than true depletion; they found no strong overall correlation between early postoperative PPBS visibility and DI once timing was taken into account [58]. In Saeki’s series, transient postoperative DI occurred in 9/15 patients despite a visible PPBS on all postoperative MRIs, and all DI resolved by 1–2 months, again illustrating that early water-balance disturbances can arise even when the bright spot is structurally intact [72]. While loss of the PPBS supports a diagnosis of central DI, the sign is neither fully sensitive nor specific and must be interpreted in the clinical context [69].

For mid-term postoperative surveillance in acromegaly, these findings translate into a pragmatic approach: reappearance or persistence of the PPBS at 3–6 months in a clinically euvolemic biochemically normal patient is reassuring, supportive indicator of preserved posterior pituitary function, but not a standalone guarantee. Temporary absence at 3 months may reflect surgical manipulation, evolving position of the posterior lobe, or transient changes in vasopressin storage, especially in the early post-op period, may resolve by 6 months, with reappearance of the PPBS. Conversely, a persistently absent PPBS at mid-term should prompt careful review of the clinical picture (polyuria, polydipsia, serum sodium/osmolality, desmopressin use) rather than automatic labeling of permanent DI. In the context of 3–6-month imaging, the PPBS should therefore be treated as one piece of a multimodal assessment, useful for supporting preserved or recovering neurohypophyseal function when present and stable, but always integrated with symptoms, biochemistry, and the broader postoperative course.

### 3.8. Cavernous Carotid Disease

**Figure 8:**
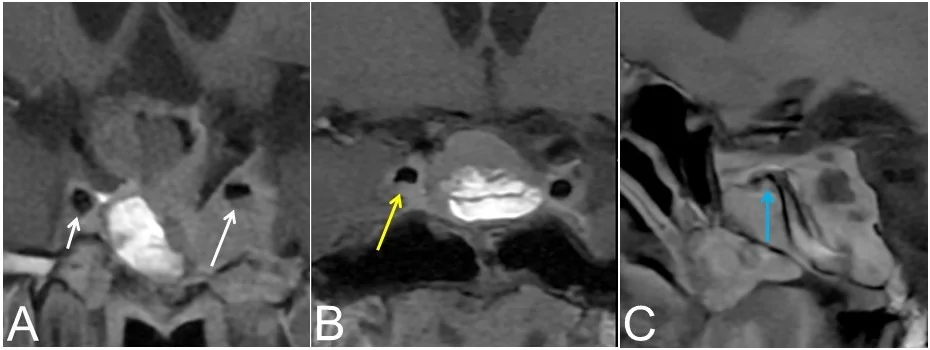
Postoperative Carotid Observations. Coronal T1WI (**A**) obtained 5 months after surgery demonstrates intraluminal disease in the left cavernous carotid artery (arrow). Comapre to the right side (short arrow) as normal reference. Coronal unenhanced T1WI (**B**) in another postoperative patient shows subtle intraluminalbulge (yellow arrow) along the inferior border of the right cavernous carotid. Sagittal post-contrast T1WI (**C**) in a different patient demonstrates intraluminal nodular enhancement inthe cavernous carotid artery (blue arrow). These examples illustrate the spectrum of postoperative cavernous carotid disease.

At 3–6 months after transsphenoidal surgery, new cavernous internal carotid artery (ICA) abnormalities on sellar MRI—such as an eccentric intraluminal filling defect, focal cavernous ICA bulging, or a nodular intraluminal enhancing focus (Figure 8)—span a spectrum from subacute mural hematoma/dissection through iatrogenic pseudoaneurysm to tumor-related vascular involvement (aneurysm developing within or adjacent to an invasive adenoma, or rarely, true intraluminal tumor). Large surgical series confirm that ICA injury during transsphenoidal surgery is rare (0.1–0.3% of cases in modern endoscopic series), but when it occurs it carries high neurological morbidity and mortality [73,74,75]. Mid-term postoperative MRI therefore has a dual role: not only assessing residual adenoma, but also screening for delayed cavernous carotid pathology, particularly in patients with acromegaly, prior cavernous sinus invasion, or re-operations—groups in whom carotid displacement and narrowing of the intercarotid distance are recognized risk factors [75,76].

Case series and reports outline the key entities and their imaging features. Pseudoaneurysms of the cavernous ICA are a well-described vascular complication of endoscopic endonasal transsphenoidal (EET) surgery, with reported incidences around 0.55–2% of EET procedures; they typically arise from a focal wall defect and may present days to weeks after surgery with recurrent epistaxis or ischemic stroke [76,77,78]. On CTA or digital subtraction angiography (DSA) they appear as saccular or fusiform outpouchings of the cavernous ICA, and on MRI as focal cavernous ICA dilation or nodular intraluminal enhancement contiguous with the arterial lumen [78,79], similar to the focal bulge and enhancing nodule in Figure 8. In the acromegaly case series, pseudoaneurysms arose from the cavernous ICA segment after otherwise uncomplicated EET, with delayed epistaxis at 1–3 weeks and successful treatment using flow-diverter stents; the authors note both the high rupture mortality (30–50%) and the particular surgical risk profile in acromegaly [76]. Subacute mural hematoma or dissection in the cavernous ICA, whether traumatic or spontaneous, produces eccentric T1-hyperintense mural thickening narrowing the flow lumen, a pattern well described in carotid dissection imaging series [80,81] and compatible with the intraluminal disease. Finally, invasive pituitary tumors can be intimately associated with cavernous ICA aneurysms or pseudoaneurysms: for example, Giorgianni et al. reported a giant prolactinoma with a cavernous ICA pseudoaneurysm embedded in the tumor, and a combined endovascular flow-diversion plus medical therapy strategy led to parallel reduction in both the adenoma and the aneurysm sac [82]. These reports reinforce that tumor invasion, carotid wall injury, and aneurysm formation frequently coexist in the cavernous sinus compartment.

At the same time, the literature illustrates how subtle carotid contour changes can be misinterpreted and how vascular and tumoral lesions can masquerade as one another. Multiple series of intrasellar and intracavernous aneurysms show that aneurysms protruding into the sella often mimic pituitary macroadenomas on routine MRI, presenting with visual symptoms or hypopituitarism and only later recognized as vascular on angiography; misdiagnosis has led to catastrophic intraoperative hemorrhage in some historical cases [83,84,85]. Conversely, in the non-postoperative setting, giant cavernous or paraclinoid aneurysms may present primarily with pituitary dysfunction and appear as sellar masses, further underscoring the overlap [86,87]. Not every minor cavernous ICA irregularity on MRI necessarily represents a pseudoaneurysm requiring treatment: high-resolution vascular imaging studies of carotid dissection show that mural hematomas can resolve with restoration of normal lumen over months, and some aneurysms discovered incidentally in association with adenomas are managed conservatively or after staged endovascular treatment [79,82,88]. Together, these data emphasize two cautionary points: (1) routine sellar MRI alone cannot reliably distinguish vascular lesions from tumor recurrence, and (2) small mural changes may reflect either benign healing (e.g., resolving dissection) or evolving high-risk pathology, so the clinical and vascular context is crucial.

For the 3–6-month postoperative MRI in acromegaly, the practical message is that any new or progressive cavernous ICA abnormality (eccentric intraluminal signal, focal bulging of the cavernous segment, or intraluminal nodular enhancement) should be explicitly recognized and treated as a potential vascular complication until proven otherwise. In this time window, persistent or enlarging focal bulges or enhancing intraluminal nodules warrant dedicated vascular imaging (CTA, MRA, and/or DSA) and multidisciplinary review with neurosurgery and interventional neuroradiology, rather than being assumed to represent residual adenoma [73,76,89]. Distinguishing vascular pathology (mural hematoma, pseudoaneurysm, carotid–cavernous fistula) from tumor recurrence or encasement is critical for patient safety and may completely change management—from endovascular repair and avoidance of re-operation through the cavernous sinus, to safe consideration of further tumor-directed surgery or radiosurgery once the vessel is secured. In short, when a cavernous carotid looks abnormal on a mid-term postoperative pituitary MRI, it should trigger formal vascular evaluation and explicit reporting, not a default assumption of tumor or benign postoperative change.

## 4. Future Directions

Future work should include a prospective, multicenter cohort of postoperative acromegaly patients (target number ≥ 200) with standardized pituitary MRI at 3 and 6 months using a harmonized protocol (pre/dynamic/post-contrast 3D T1, high-resolution T2, DWI). Radiologists should score eight predefined patterns—packing material, late-phase enhancing nodule, thin rim enhancement, cavernous sinus tissue, stalk deviation vs. thickening, floor-based granulation tissue, PPBS absence/reappearance, and cavernous carotid abnormalities—using explicit, reproducible criteria (e.g., rim thickness in mm; nodule size ≥ 3–4 mm; stalk diameter cutoffs; quantitative T2 signal ratios; ADC thresholds). These imaging scores should be correlated with IGF-1/GH status at 6 and 12 months and with surgical findings, to generate per-sign sensitivity, specificity, PPV, NPV, and accuracy for active disease. Parallel radiomics and artificial intelligence analyses should test whether texture and shape features of nodules, granulation, and cavernous sinus tissue outperform visual reads. The ultimate goal is to derive and externally validate a simple, point-based 3–6-month postoperative MRI score that stratifies acromegaly patients into low, intermediate, and high risk for clinically relevant residual or recurrent disease.

## 5. Conclusions

**Figure 9:**
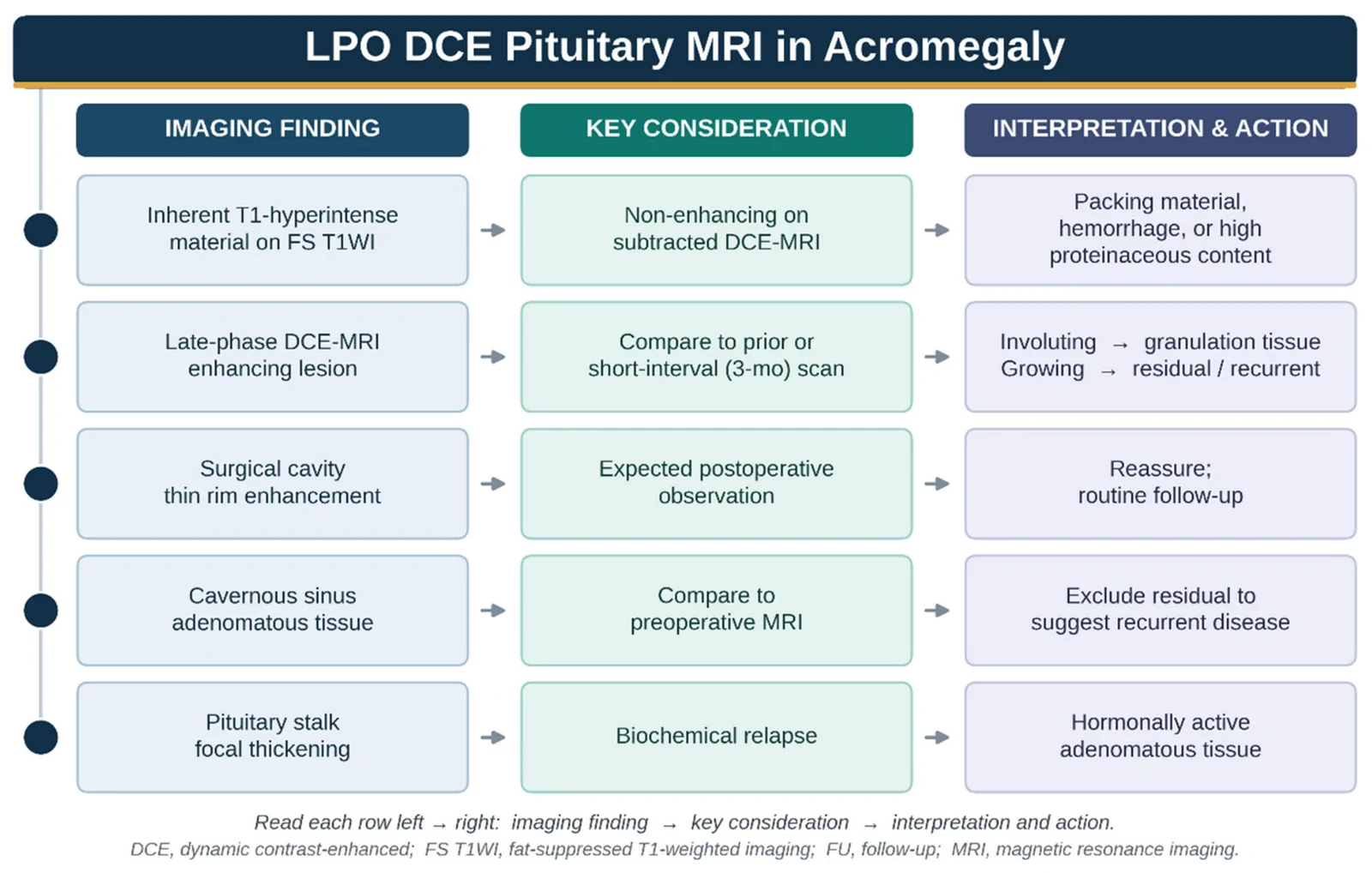
Late Postoperative Pituitary MRI Patterns (following transsphenoidal surgery for acromegaly).

In conclusion, late postoperative (3–6-month) pituitary MRI in acromegaly can be interpreted in a structured, pattern-based approach that is captured in our accompanying flowchart (Figure 9). A discrete nodular enhancement in the resection bed or new mass effect since the prior scan favors residual or recurrent adenoma. Inherent T1-hyperintense non-enhancing material along the sellar floor or wall usually reflects fat, blood products, or packing material. Thin smooth rim enhancement around a cystic cavity without a solid component is most consistent with surgical fossa granulation tissue. Soft tissue in the cavernous sinus, progressive stalk thickening, and new cavernosal carotid artery abnormalities are high-risk imaging findings that warrant multidisciplinary review and should be followed at short interval. By explicitly linking these reproducible imaging patterns with IGF-1/GH status and the corresponding management pathways, radiologists and endocrinologists can more reliably distinguish expected postoperative changes from clinically relevant residual or recurrent disease in daily practice.

## Figures and Tables

**Figure 3:**
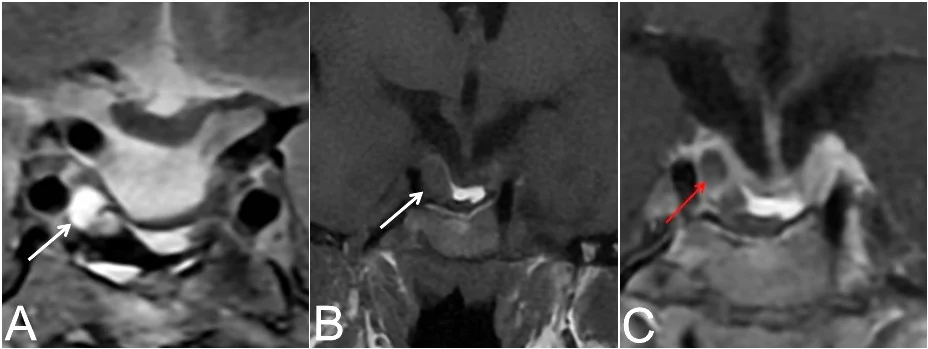
Surgical Bed Faint Enhancement. 3 months post transsphenoidal surgery, with multiple normal range IGF-1 levels, was referred for imaging surveillance. Clinical exams were within normal limits. Coronal T2WI (**A**) shows a high signal structure in the surgical bed (white arrow), corresponding to a low-signal are on coronal T1WI (**B**). Post-contrast coronal T1WI (**C**) demonstrates faintly enhancing internal content (red arrow) without prominnet nodularity or thick septa.

**Table 1:** Pituitary DCE MRI protocol.

Sequence	TR (ms)	TE (ms)	FA (°)	ST (mm)	FOV	Temporal Resolution	BW (kHz)	Scan Time
Sagittal T1w FSE	350–400	Min full	160	2.5	150 mm	-	41.67	1:40
Coronal T2w FSE	2000	102	160	2.5	150 mm	-	41.67	1:30
Sagittal T2w FSE	2600	102	160	2.5	150 mm	-	41.67	1:20
Coronal T1 DYN	350–400	Min full	160	2.5	150 mm	22s	41.67	3:10
Coronal T1w F.S+C	500–700	Min full	160	2.5	150 mm	-	41.67	1:50
Sagittal T1w FSE+C	350–400	Min full	160	2.5	150 mm	-	41.67	1:40

Abbreviations used in the table: TR: Time of repetition; ms: milliseconds; TE: echo time; FA: flip angle; ST: slice thickness; BW: bandwidth.

## Data Availability

Source imaging files for the cases illustrated in this review can be accessed through the corresponding author upon reasonable request.
